# Herbal Composition LI73014F2 Alleviates Articular Cartilage Damage and Inflammatory Response in Monosodium Iodoacetate-Induced Osteoarthritis in Rats

**DOI:** 10.3390/molecules25225467

**Published:** 2020-11-23

**Authors:** Hae Lim Kim, Hae Jin Lee, Dong-Ryung Lee, Bong-Keun Choi, Seung Hwan Yang

**Affiliations:** 1 Department of Biotechnology, Chonnam National University, Yeosu 59626, Korea; ics1357@naver.com (H.L.K.); haecutejin@naver.com (H.J.L.); 2 Nutrapharm Tech, Jungwon-gu, Seongnam, Gyunggi 13201, Korea; drlee@nutrapharm.co.kr (D.-R.L.); cbcbcbk@nutrapharm.co.kr (B.-K.C.)

**Keywords:** *Boswellia serrata* extracts, *Terminalia chebula* fruit extracts, *Curcuma longa* rhizome extracts, LI73014F2, osteoarthritis, cartilage degradation, inflammatory response

## Abstract

The aim of this study was to determine the anti-osteoarthritic effects of LI73014F2, which consists of *Terminalia chebula* fruit, *Curcuma longa* rhizome, and *Boswellia serrata* gum resin in a 2:1:2 ratio, in the monosodium iodoacetate (MIA)-induced osteoarthritis (OA) rat model. LI73014F2 was orally administered once per day for three weeks. Weight-bearing distribution and arthritis index (AI) were measured once per week to confirm the OA symptoms. Synovial membrane, proteoglycan layer, and cartilage damage were investigated by histological examination, while synovial fluid interleukin-1β level was analyzed using a commercial kit. Levels of pro-inflammatory mediators/cytokines and matrix metalloproteinases (MMPs) in the cartilage tissues were investigated to confirm the anti-osteoarthritic effects of LI73014F2. LI73014F2 significantly inhibited the MIA-induced increase in OA symptoms, synovial fluid cytokine, cartilage damage, and expression levels of pro-inflammatory mediators/cytokines and MMPs in the articular cartilage. These results suggest that LI73014F2 exerts anti-osteoarthritic effects by regulating inflammatory cytokines and MMPs in MIA-induced OA rats.

## 1. Introduction

Osteoarthritis (OA) is a common joint disorder that is characterized by chronic pain, joint inflammation, swelling, and movement limitation [1,2]. OA is the most common form of arthritis, which causes tremendous economic burden and disrupts the quality of life. It can be worsened by genetic factors, an overweight status, and post-traumatic arthritis [3]. In particular, age is a major cause of increased OA incidence in people over 65 years old [4]. Medicines or functional foods have generally been used to treat OA; however, pharmacological treatments of OA are limited in their effectiveness. Management of the condition typically focuses on reducing pain and inflammation using non-steroidal anti-inflammatory drugs (NSAIDs), including acetaminophen, ibuprofen, naproxen, and indomethacin [5]. However, long-term intake of NSAIDs has adverse effects on the gastrointestinal and cardiovascular systems. The efficacy of functional foods such as glucosamine and chondroitin sulfate remain insufficient [4,5].

The pathogenesis of OA involves pro-inflammatory cytokines and matrix metalloproteinases (MMPs) that play an important role in the severity of OA [6,7]. Pro-inflammatory cytokines induce the production of MMPs, which enzymatically degrade cartilaginous matrix substances leading to cartilage breakdown. Mechanical force damages the synovial cell membrane and increases membrane phospholipid release, which is then converted to arachidonic acid, and induces the cyclooxygenase (COX) and 5-lipoxygenase (5-LOX) pathways. This enzyme pathway activation promotes the production of prostaglandins and leukotrienes [8]. It has been previously reported that 5-LOX causes synovial deterioration, and leukotriene B_4_ (LTB_4_) is a potent inflammatory metabolite that stimulates tumor necrosis factor-alpha (TNF-α) and interleukin-1β (IL-1β) production from human OA synovial explants [9,10].

LI73014F2 is a novel composition that contains extracts of *Terminalia chebula* fruit, *Curcuma longa* rhizome, and *Boswellia serrata* gum resin (in a 2:1:2 ratio). Extracts of *T. chebula*, *C. longa*, and *B. serrata* have traditionally been used as Ayurvedic medicine to treat various symptoms and disorders. *Boswellia* extract is used to treat asthma, rheumatism, dysentery, skin ailments, ulcers, and for blood purification; it is safe for consumption and efficacious. It contains compounds such as 3-O-acetyl-11-keto-β-boswellic acid (AKBA), 11-keto-β-boswellic acid, and β-boswellic acid [11,12]. *Curcuma longa*, which contains curcumin, demethoxycurcumin, bisdemethoxycurcumin, and turmeric acid, has been proven to be safe and is in use for a long time [13]. Known as gang-hwang in Korea, it is known to have strong anti-inflammatory and anti-oxidant activities [14,15]. *Terminalia chebula* is a widely growing evergreen tree in India and Southeast Asia, known as Ga-ja in Korea. Anti-oxidant, cytoprotective, and anti-inflammatory effects have been demonstrated for this botanical, which is composed of gallic acid, chebulagic acid, chebulinic acid, and punicalagin, among other bioactive compounds [16,17]. In previous studies, synergic effects were identified by comparing the 5-LOX activity of a single extract with the LI73014F2 formulation; these studies have also demonstrated its anti-osteoarthritic effects in human chondrocytes [8,17]. However, there have been no studies on the efficacy and related mechanisms of LI73014F2 in MIA-induced OA rat models.

In the present study, we further investigated the potential applications of LI73014F2 based on its synergistic activity and evaluated its protective effects on articular cartilage in an MIA-induced OA rat model.

## 2. Results

### 2.1. Composition of LI73014F2

To confirm the composition of LI73014F2, we performed high-performance liquid chromatography analysis. Based on a previous study [8], we established the standard compound and specified the content range (data not shown). Gallic acid is a phytochemical marker for *T. chebula* fruit raw material, while total curcuminoids (curcumin, demethoxycurcumin, and bismethoxycurcumin) and AKBA are unique phytochemical markers for *C. longa* rhizome and *B. serrata* resin raw materials, respectively. The levels of gallic acid, total curcuminoids (curcumin, demethoxycurcumin, and bismethoxycurcumin), and AKBA in LI73014F2 were found to be approximately 18, 35, and 9 mg/g, respectively.

### 2.2. Effects of LI73014F2 on Changes in Body Weight in Monosodium Iodoacetate (MIA)-Induced Osteoarthritis in Rats

**Figure 1:**
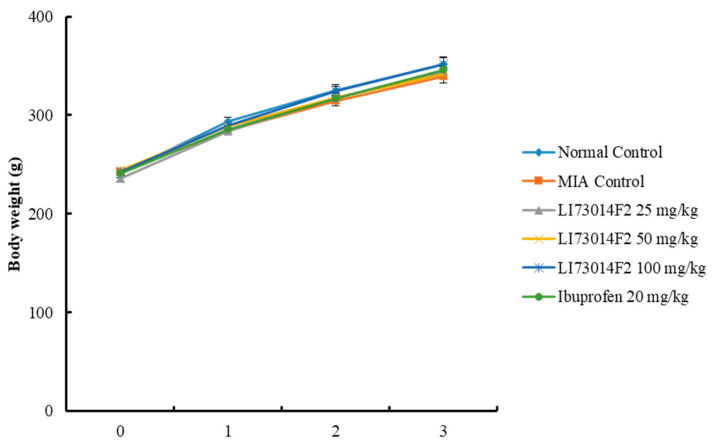
Effects of LI73014F2 on changes in body weight in monosodium iodoacetate (MIA)-induced osteoarthritis in rats. Body weight was evaluated once a week for 3 weeks and data are presented as mean ± SEM (*n* = 8/group). No significant difference was detected between all the groups.

The effects of LI73014F2 on the body weight of all the animals was noted once a week and the changes were observed for three weeks. There was no statistically significant difference between any of the groups (Figure 1) for three weeks, indicating that LI73014F2 did not affect the body weight.

### 2.3. Effects of LI73014F2 on the Hind Paw Weight-Bearing Distribution for 21 Days in MIA-Induced Osteoarthritis in Rats

To assess OA progression, we evaluated the hind paw weight-bearing capabilities and confirmed the ratio of weight distribution between the left (MIA-injected) and right (healthy) limbs using an incapacitance tester on days 0, 7, 14, and 21. The ratio of weight distribution for the MIA-treated group was significantly lower than that for the normal control group on day 7 and this significant difference (*p* < 0.01) was maintained until day 21. However, the ratios of the LI73014F2- (22%, 25%, and 24% for doses 25, 50, and 100 mg/kg, respectively) and ibuprofen (20%)-treated groups increased as compared to those of the MIA-treated group on day 7. In particular, the weight distribution of 25 mg/kg (42.67 ± 1.02), 50 mg/kg (43.19 ± 0.96), and 100 mg/kg (44.95 ± 0.89) LI73014F2-treated groups returned to the normal level and showed similar results to those of the ibuprofen-treated group (45.35 ± 0.66) on day 21 (Table 1). These results indicate that LI73014F2 might alleviate the pain symptoms of OA.

### 2.4. Effects of LI73014F2 on Arthritis Index (AI) for 21 Days in MIA-Induced Osteoarthritis in Rats

To identify global OA symptoms such as swelling and limping, we observed all the animals weekly. Animals in the MIA-treated group showed a higher arthritis index (AI) score including swelling and limping score compared to all the other groups; the index of the MIA-treated group gradually decreased as time passed (Table 2). Rats treated with 25 mg/kg (*p* < 0.01), 50 mg/kg (*p* < 0.01), and 100 mg/kg (*p* < 0.01) LI73014F2 had markedly reduced AI scores after day 14, when in comparison with the MIA-treated group. Furthermore, after day 21, administration with 50 mg/kg LI73014F2 decreased the AI score to a level similar to that of ibuprofen, the positive control. Thus, we confirmed that increased AI scores due to OA were significantly reduced upon LI73014F2 treatment.

### 2.5. Effects of LI73014F2 on Synovial Fluid Levels of Inflammatory Factors in MIA-Induced Osteoarthritis in Rats

**Figure 2:**
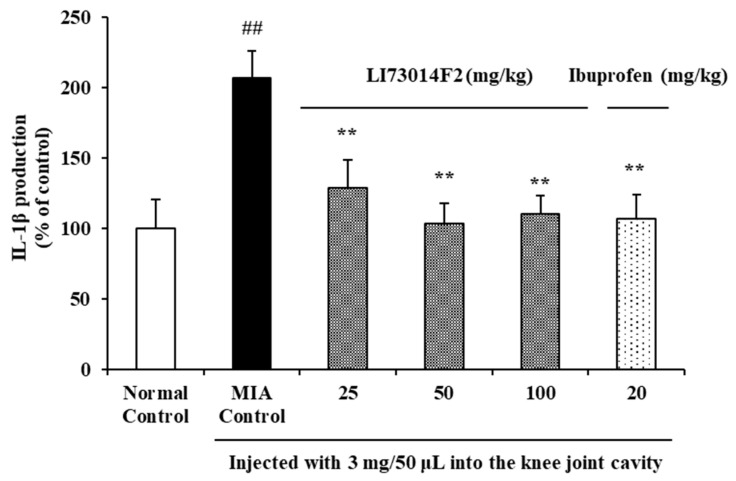
Effects of LI73014F2 on synovial fluid levels of inflammatory factors in MIA-induced osteoarthritis in rats. The collected synovial fluid was analyzed for the levels of IL-1β using commercial ELISA kits. Data are expressed as mean ± SEM (*n* = 8/group). ** *p* < 0.01, compared to the MIA-induced control group; ^##^ *p* < 0.01, compared to the non-MIA-induced control group.

To evaluate the effects of LI73014F2 on IL-1β levels of MIA-induced OA rats, synovial fluid was collected and the IL-1β levels in it were measured. IL-1β level was significantly higher in rats injected with MIA than in the normal control group. As shown in Figure 2, IL-1β level reduced by 38%, 50%, and 48% following treatment with 25 mg/kg, 50 mg/kg, and 100 mg/kg LI73014F2, respectively than MIA-treated group (*p* < 0.01). The concentration of IL-1β in the synovial fluid of animals in the ibuprofen-treated group was significantly lower (by 49%, *p* < 0.01) than in the MIA-treated group. Animals treated with 50 or 100 mg/kg LI73014F2, or with ibuprofen had concentrations of synovial fluid IL-1β that were similar to those in the normal control group.

### 2.6. Effects of LI73014F2 on the Expression Levels of Inflammation-Related Proteins in Articular Cartilage

**Figure 3:**
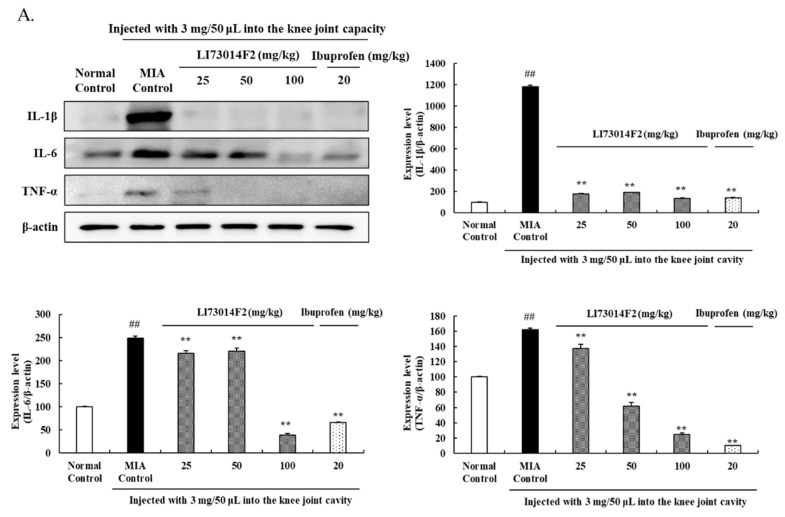

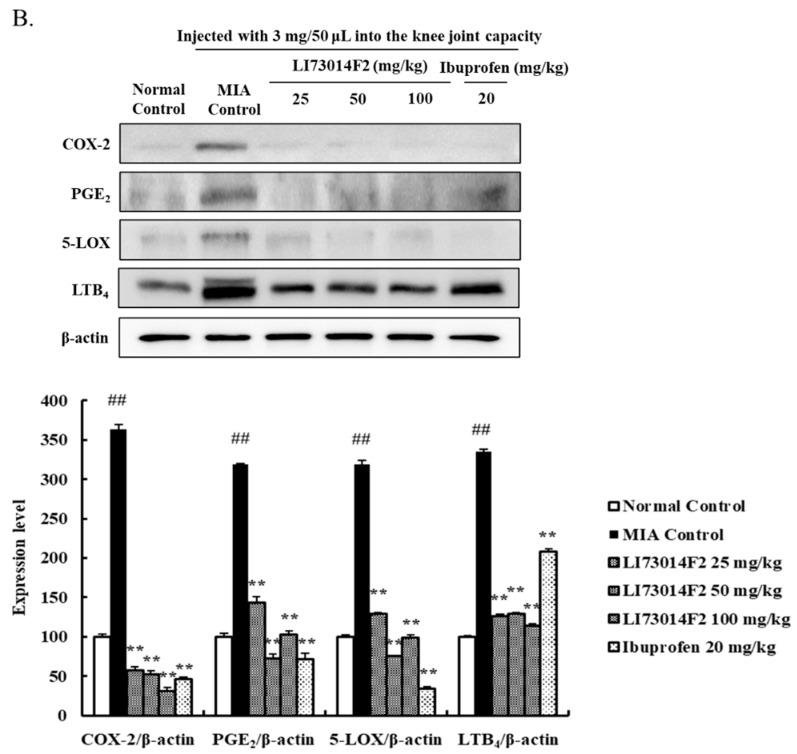
Effects of LI73014F2 on the expression levels of inflammation-related proteins in articular cartilage. The expression levels of (**A**) pro-inflammatory cytokines interleukin-1β (IL-1β), IL-6, and tumor necrosis factor-alpha (TNF-α), and (**B**) inflammatory mediators cyclooxygenase-2 (COX-2), prostaglandin E2 (PGE_2_), 5-lipoxygenase (5-LOX), and leukotriene B_4_ (LTB_4_) were measured using Western blot analysis; density of the protein bands was quantified and calculated using ImageJ software. Protein expression levels were normalized to those of β-actin and are expressed as mean ± SEM of independent experiments (*n* = 3/group). ** *p* < 0.01, compared to the MIA-induced control group; ^##^ *p* < 0.01, compared to the non-MIA-induced control group.

To demonstrate the effect of LI73014F2 on OA-generated inflammation, the protein expression levels of COX-2, PGE_2_, 5-LOX, LTB_4_, IL-1β, IL-6, and TNF-α were analyzed in the cartilage tissue. MIA-induced OA significantly increased the protein levels of COX-2, PGE_2_, 5-LOX, LTB_4_, IL-1β, IL-6, and TNF-α. As shown in Figure 3A, the 25 mg/kg LI73014F2-treated group reduced the expression levels of IL-1β, IL-6, and TNF-α by 79%, 13%, and 15%, respectively, when in comparison with the MIA-treated group. Treatment with 50 mg/kg LI73014F2 significantly decreased (*p* < 0.01 for all conditions) the expression of IL-1β, IL-6, and TNF-α by 84%, 11%, and 67%, respectively, with respect to the expression levels in the MIA-treated group. Additionally, 100 mg/kg LI73014F2 significantly reduced (*p* < 0.01) the 1L-1β, 1L-6, and TNF-α expression levels to 89%, 84%, and 38%, respectively, as compared to those observed in the MIA-treated group.

As shown in Figure 3B, COX-2 protein levels were significantly lower in the groups treated with LI73014F2 (*p* < 0.01) and ibuprofen (*p* < 0.01). Treatment with 25, 50, and 100 mg/kg LI73014F2 lowered the PGE_2_ expression by 55%, 77%, and 68%, respectively (*p* < 0.01 for all conditions), whereas ibuprofen decreased the PGE_2_ expression by 77% (*p* < 0.01). When compared to the MIA-induced group, the 5-LOX expression level dose-dependently reduced by 60%, 78%, and 69% upon treatment with 25, 50, and 100 mg/kg LI73014F2, respectively (*p* < 0.01 for all conditions), while ibuprofen treatment significantly reduced (*p* < 0.01) the 5-LOX expression by 90%. In addition, LTB_4_ expression in the 25, 50, and 100 mg/kg LI73014F2-treated groups significantly reduced (*p* < 0.01 for all conditions) by 63%, 61%, and 66%, respectively, while ibuprofen administration reduced the LTB_4_ expression by 38% (*p* < 0.01).

### 2.7. Effects of LI73014F2 on Histological Evaluation of Joint Activity in MIA-Induced Osteoarthritis in Rats

**Figure 4:**
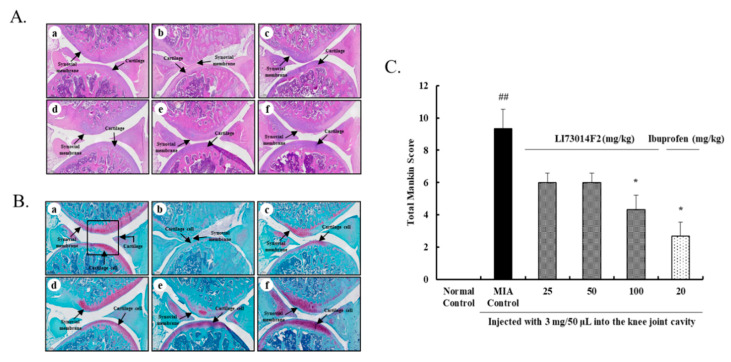
Effects of LI73014F2 on the histological evaluation of joint activity in MIA-induced osteoarthritis in rats. (**A**) Cartilage and synovial membrane in knee joints were stained with hematoxylin and eosin (H&E), (**B**) cartilage cell and proteoglycan layer in knee joints stained with Safranin O, and (**C**) graded on a 0–13 scale using the Mankin scoring system. (a) Normal Control group, (b) MIA Control group, (c) MIA + LI73014F2 25 mg/kg, (d) MIA + LI73014F2 50 mg/kg, (e) MIA + LI73014F2 100 mg/kg, and (f) MIA + ibuprofen 20 mg/kg. Data are expressed as mean ± SEM (*n* = 5/group). * *p* < 0.05, compared to the MIA-induced control group; ^##^ *p* < 0.01, compared to the non-MIA-induced control group.

To confirm the morphological changes and severity of the articular damage, hematoxylin and eosin (H&E) and Safranin-O staining were performed in the MIA-induced OA rats. H&E staining analysis showed that the MIA-treated group exhibited significant changes in cartilage, synovial membrane, and fibrous tissue. However, administration of LI73014F2 or ibuprofen effectively alleviated these structural morphological changes in the articular cartilages as compared to the MIA-treated group (Figure 4A). In addition, we stained the cartilage cell and proteoglycan layer with Safranin O to evaluate cartilage degradation (Figure 4B). In the MIA-treated group, the red-stained normal cartilage was destroyed by MIA and the proteoglycan layer disappeared. In contrast, the proteoglycan layer was clearly seen in the groups treated with 50 and 100 mg/kg LI73014F2 and ibuprofen. Furthermore, the Mankin scoring system was used to score the severity of the OA lesions, as shown in Figure 4C. Compared to that in the MIA-treated group, the Mankin score was lower by 36% in the 50 mg/kg LI73014F2-treated group, while it was significantly lower in the 100 mg/kg LI73014F2- and 20 mg/kg ibuprofen-treated groups (by 54% and 71%, respectively).

### 2.8. Effects of LI73014F2 on Expression Levels of MMPs in Articular Cartilage

**Figure 5:**
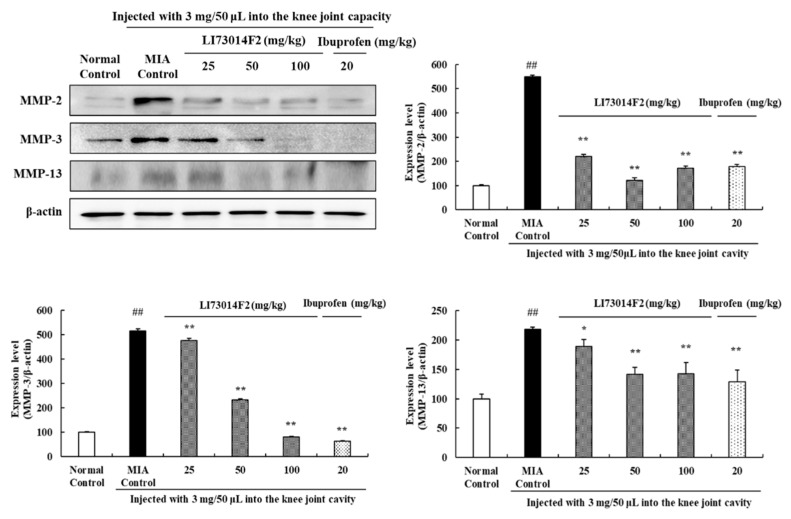
Effects of LI73014F2 on the expression levels of matrix metalloproteinases (MMPs) in articular cartilage. The expression levels of MMP-2, MMP-3, and MMP-9 were determined using Western blot analysis; density of the protein bands was quantified and calculated using ImageJ software. Protein expression levels were normalized to those of β-actin and are expressed as mean ± SEM of independent experiments (*n* = 3/group). * *p* < 0.05 and ** *p* < 0.01, compared to the MIA-induced control group; ^##^ *p* < 0.01, compared to the non-MIA-induced control group.

The protein levels of MMP-2, MMP-3, and MMP-13 were evaluated to investigate the effect of LI73014F2 on MMPs in the cartilage tissues of OA-induced rats. As shown in Figure 5, MIA injection significantly enhanced MMP-2, MMP-3, and MMP-13 expression in comparison with the normal control group. Treatment with 25, 50, and 100 mg/kg LI73014F2 and ibuprofen significantly reduced (*p* < 0.01 for all conditions) MMP-2 levels, compared to those in the MIA-treated group. In addition, there was a dose-dependent decrease in the levels of MMP-3 upon administration of 25 mg/kg (by 7%) and 50 mg/kg (by 54%) LI73014F2, as compared to the MIA-treated group. Moreover, treatment with 100 mg/kg (by 84%) LI73014F2 and ibuprofen (by 87%) also significantly inhibited the MMP-3 expression. Moreover, the OA-induced increase in the expression levels of MMP-13 was significantly decreased upon administration of 50 mg/kg (by 35%) and 100 mg/kg (by 34%) LI73014F2, as well as of ibuprofen (by 40%).

## 3. Discussion

Osteoarthritis (OA) is a painful and debilitating disease characterized by progressive loss of articular cartilage and mild inflammation of the tissue around the joint. OA patients undergo persistent arthritic pain and display increased sensitivity to pressure in the joint, making mobility difficult [18]. Therefore, OA management is focused on alleviating pain, increasing joint mobility, improving joint strength, and minimizing the disabling effects of the disease [19]. In the present study, we investigated the anti-osteoarthritic effects of a novel botanical ingredient using an MIA-induced OA rat model. Monosodium iodoacetate (MIA) is an inhibitor of glyceraldehyde-3-phosphate dehydrogenase, interferes with cellular glycolysis and leads to cartilage degeneration with increased inflammation [20,21]. Inflammatory stimuli lead to cytokine release and complex biochemical and mechanical interplay with other mediators to induce OA and promote disease symptoms such as pain, swelling, and stiffness [22,23]. MIA-induced OA is a well-established model to evaluate the efficacy of potential modulators of OA. The model mimics symptoms observed in patients with OA and has been applied to many studies related to OA [22].

Weight-bearing distribution is used to measure the pain and index of joint discomfort. A decrease in its value in the affected limb upon MIA injection is indicative of joint pain [24,25]. The present study showed that treatment with 50 mg/kg LI73014F2 significantly increased the weight-bearing distribution, which may present the symptom of alleviation in pain. Furthermore, global OA symptoms such as swelling and limping were confirmed by observing knee joint swelling and walking patterns. This means that LI73014F2 administration showed significant effects in alleviating OA symptoms and pain-related behaviors. The improvement in OA symptoms was related with decreased levels of inflammatory cytokines, which are known to exacerbate pain and joint destruction [2].

The pro-inflammatory mediator PGE_2_ is produced in the inflamed synovium and plays an important role in the development of inflammation in OA [26]. COX-2 overproduction directly stimulates high PGE_2_ production during inflammation. A significant amount of PGE_2_ causes inflammatory reactions and induces pain and degeneration associated with OA [23,26]. In addition, the 5-LOX pathway plays an important role in early OA development and affects disease progression by damaging the synovial membrane. LTB_4_ can increase microvascular permeability and also increase the production and release of cytokines such as TNF-α and IL-1β [8,27].

Moreover, inflammatory cytokines can be detected in the synovial fluid from OA patients, suggesting that they play an important role in promoting the catabolic process in OA, thus causing cartilage degradation [22,23,28,29]. Among these, IL-1β is one of the most well studied cytokines that is highly expressed in the cartilage and synovial tissue. It blocks the synthesis of extracellular matrix (ECM) and contributes to the enhancement of cartilage breakdown by activating collagenases, especially MMP-3 and MMP-13 [23]. IL-6 is another well-known pro-inflammatory cytokine involved in the pathophysiology of OA. It induces cartilage damage and destruction and has been associated with hyperalgesia and hypersensitivity in joint tissues [23,30]. TNF-α is a potent pro-inflammatory cytokine that is responsible for initiating joint destruction. It initiates cascades of inflammatory reactions via the production of IL-1β and IL-6 and inhibits proteoglycan synthesis, resulting in the loss of cartilage [30,31]. Therefore, we confirmed the protein levels of pro-inflammatory mediators (COX-2, PGE_2_, 5-LOX, and LTB_4_) in the cartilage tissue as well as pro-inflammatory cytokines (IL-1β, IL-6, and TNF-α) in the synovial fluid of MIA-induced OA rats treated with LI73014F2 in order to investigate its anti-inflammatory effects. We observed that LI73014F2 application could reduce the MIA-induced increase in cytokines, similar to ibuprofen. These results indicate that LI73014F2 can decrease inflammatory response and mediators, subsequently reducing cartilage damage.

Articular cartilage damage starts when proteoglycan, the main component of the ECM, is broken down by MMPs [32]. In the present study, we observed the histological changes using H&E and Safranin-O staining of the articular cartilage. We confirmed the damage to the synovial membrane and cartilage along with the proteoglycan destruction in MIA-induced OA rats. Administration of 50 mg/kg LI73014F2 effectively inhibited the synovial membrane damage and cartilage degradation. These results support the conclusion that LI73014F2 administration mediates anti-osteoarthritic effects by inhibiting cartilage damage and proteoglycan layer destruction.

MMPs are important proteases involved in tissue remodeling including ECM degradation and can be increased by pro-inflammatory cytokines [33]. MMP-2 is a gelatinase that degrades a broad range of collagen and proteoglycan [17] and has been reported to cause an increase in the OA cartilage [34]. MMP-3 is secreted from chondrocytes and synovial cells and cleaves the matrix components, causing fibrillation, erosion, and cracking of the cartilage tissue [35]. MMP-13 plays a key role in collagen and proteoglycan degradation and is known to increase during the early OA process [35,36]. We analyzed MMP expression to assess the anti-osteoarthritis effects of LI73014F2 and showed that a 50 mg/kg dose of the compound significantly reduced MMP-2, MMP-3, and MMP-13 levels. These results indicate that the cartilage damage is inhibited by the regulation of MMP levels.

In the present study, we confirmed the anti-osteoarthritic effects of LI73014F2 in MIA-induced OA rats. LI73014F2 showed anti-inflammatory effect by reducing the levels of pro-inflammatory mediators and pro-inflammatory cytokines. In addition, it suppressed the destruction of the synovial membrane and articular cartilage by decreasing the expression levels of MMPs, thereby suppressing the progression of OA. These results, together with the clinical data that show efficacy [8], demonstrate that LI73014F2 may be beneficial for the management of OA in humans.

## 4. Materials and Methods

### 4.1. Sample Preparation and Component Analysis

LI73014F2 is a synergic composition comprising an aqueous extract of *T. chebula* fruit, alcoholic extract of *C. longa* rhizome, and *B. serrata* gum resin (in a 2:1:2 ratio). LI73014F2 was prepared by the same method as previously reported [8,17]. This ingredient is produced by Laila Nutraceuticals (Vijayawada, India) and sold as Flexir^®^ by PLT Health Solutions (Morristown, NJ, USA). The finished formulation was standardized to approximately 1.8% gallic acid, 3.5% total curcuminoids (curcumin, demethoxycurcumin, and bismethoxycurcumin), and 0.9% 3-O-acety-11-keto-b-boswellic acid (AKBA).

### 4.2. Animals

Sixty 6-week-old male Sprague Dawley rats (130–190 g) were purchased from Samtako Bio Inc. (Osan, Korea). All animals were acclimatized for seven days and healthy animals were selected and used for experimentation. The experiment was conducted under temperature- (22 ± 2 °C, humidity 50% ± 1%) and light (12 h light/dark cycle)-controlled conditions. The animals had ad libitum access to sterile food and water. The study was carried out in accordance with the National Guidelines for the Care and Use of Laboratory Animals approved by the Animal Ethics Committee (permission number: IV-RB-02-1910-21 of INVIVO Co., Ltd. (Chungnam, Korea)). We recorded changes in body weight once a week and observed changes in feed and water intake.

### 4.3. MIA-Induced OA and Drug Administration

For the induction of OA, the left knee of the rats was shaved and disinfected with 70% alcohol followed by a single injection of 0.9% sodium chloride 50 μL containing 3 mg MIA into the synovial cavity using a 1 mL Insulin Syringe (BD Medical-Diabetes Care, Franklin Lakes, NJ, USA). After 3 days, the rats were randomly divided into 6 groups (*n* = 8 rats/group) including (1) non-MIA-induced control + vehicle, (2) MIA-induced control + vehicle, (3) MIA + 25 mg/kg LI73014F2, (4) MIA + 50 mg/kg LI73014F2, (5) MIA + 100 mg/kg LI73014F2, and (6) MIA + 20 mg/kg ibuprofen. The test compounds were dissolved in 0.5% carboxymethyl cellulose sodium salt (CMC-Na) and orally administrated once a day for 3 weeks.

### 4.4. Progression of OA and Hind Paw Weight-Bearing Distribution

At 0, 7, 14, and 21 days after test substance treatment, all rats were allowed to move freely in the cage, and their knee joint swelling and walking patterns such as gait disturbance were carefully evaluated. Swelling and limping were classified as no change (0), mild (1), moderate (2), and severe (3) and represented as arthritis index (AI) score [37,38]. The same trained evaluator, who was blinded to the treatment throughout the study period, conducted all the assessments.

After OA induction, the original balance in the weight-bearing capabilities of the hind paws was disrupted. To evaluate changes in weight-bearing tolerance, the rats were carefully placed in a measuring chamber of an incapacitance meter tester (IITC Life Science, Woodland Hills, CA, USA) and the weight-bearing force exerted by each hind limb was averaged over a 10 s period. Percent weight-bearing distribution of the left hind paw was calculated using the following equation: %weight distribution of left hind paw = weight on left hind limb/(weight on right hind limb + weight on left hind limb) × 100 [3,38].

### 4.5. Synovial Fluid Analysis

After 3 weeks of treatment, the rats were anesthetized and the synovial fluid was collected from the injected joint. The skin above the knee joint was opened transversally using a scalpel, the ligament above the joint was sectioned, and the cavity was rinsed 4 times with 100 μL sterile PBS. The fluid was centrifuged at 4 °C and the supernatant was analyzed for biochemical mediators. Level of IL-1β was determined using commercial kits (Chondrex, Seattle, WA, USA) following the manufacturer’s instructions.

### 4.6. Joint Histological Examination

To confirm the effect of LI73014F2 on cartilage degeneration in the knee joints, we assessed the histological changes in the MIA-induced OA rat model. Following sacrifice at the end of the study, the left knee joint was resected, fixed in 10% formalin (Sigma-Aldrich, St Louis, MO, USA) for 24 h at 4 °C, and decalcified with 5% hydrochloric acid (Sigma-Aldrich) for 4 days at 4 °C. Following decalcification, the specimens were dehydrated in graded acetone and embedded in paraffin. The paraffin-embedded left knee joints were sliced along the sagittal axis to a thickness of 5 μm. Five-micrometer thick sections were stained using hematoxylin and eosin (H&E) (Sigma-Aldrich) to confirm the change of synovial membrane and cartilage in the knee joint. Five-micrometer thick sections were stained as well with Safranin O/Fast Green (Sigma-Aldrich) to determine whether cartilage tissue was damaged. All stained slides were scanned using Motic EasyScan (Meyer Instrument, Houston, TX, USA) and were evaluated and graded on a 0–13 scale by a double-blinded observer, according to the Mankin scoring system [22,39].

### 4.7. Western Blotting

Cartilage tissue was removed and washed three times with ice-cold PBS. Tissues were briefly pulverized in liquid nitrogen and total protein was extracted using RIPA buffer containing 50 mM Tris-HCl pH 7.4, 150 mM NaCl, 1 mM ethylenediaminetetraacetic acid, 1% Triton X-100, 1% sodium deoxycholate, 0.1% sodium dodecyl sulfate, 1 mM phenylmethylsulfonyl fluoride, and 1% protease inhibitor cocktail (Roche, Mannheim, Germany); the lysate was subsequently centrifuged at 10,000× *g* for 15 min at 4 °C. The protein concentration in the supernatant was determined using a BCA Protein Assay Kit (Thermo Fisher Scientific, San Jose, CA, USA). The proteins were separated using 10% SDS-PAGE and then transferred to polyvinylidene difluoride membranes (Millipore Corp., Bedford, MA, USA). The membranes were initially blocked using 5% skimmed milk in Tris-buffered saline containing 0.1% Tween-20 for 30 min. Next, they were incubated with specific primary antibodies against β-actin, COX-2 (1:1000 dilution; Cell Signaling Technology Inc., Danvers, MA, USA), prostaglandin E2 (PGE_2_), 5-LOX, IL-1β, TNF-α, IL-6, MMP-2, MMP-3, MMP-13 (1:1000 dilutions; Abcam, Cambridge, MA, USA), and LTB_4_ (1:500 dilution; Enzo Life Sciences, Farmingdale, NY, USA) overnight at 4 °C. The membranes were then incubated in the corresponding horseradish peroxidase-conjugated anti-rabbit, anti-mouse immunoglobulin G (1:10,000 dilution; GenDEPOT, Barker, TX, USA) for 1 h at 23 °C. Detection was performed using the ECL system (Atto, Tokyo, Japan). The intensity of the bands on the membrane was detected using Image-Pro Plus software (6.0 version; Media Cybernetics Inc., Rockville, MD, USA).

### 4.8. Statistical Analysis

Data were expressed as mean ± standard error of the mean (SEM) and assessed using SPSS (version 22.0, SPSS Inc., Chicago, IL, USA). Different treatment groups were compared using the Student’s *t*-test and one-way analysis of variance, followed by multiple comparisons correction using Dunnett’s post-hoc test with Origin 7.0 software (OriginLab, Northampton, MA, USA). *p* < 0.05 and *p* < 0.01 were considered to indicate a statistically significant difference.

## 5. Conclusions

Injection of MIA induces degenerative changes in the articular cartilage and promotes pain symptoms and joint discomfort by increasing the levels of inflammatory cytokines and MMPs. We used this model to evaluate the efficacy of a novel botanical ingredient, LI73014F2, and showed that treatment with it decreased the weight-bearing distribution and AI scores of MIA-induced OA in rats. LI73014F2 administration also reduced the IL-1β level in the synovial fluid. Levels of the pro-inflammatory mediators including COX-2, PGE_2_, 5-LOX, and LTB_4_ and pro-inflammatory cytokines including IL-1β, IL-6, and TNF-α decreased upon administration of LI73014F2. Histopathological examination of the synovial membrane showed that LI73014F2 administration attenuated cartilage destruction. LI73014F2 treatment inhibited the pain, joint discomfort, and cartilage degradation by decreasing MMP-2, MMP-3, and MMP-13 levels, similar to ibuprofen treatment. Taken together, these results indicate that LI73014F2, at the dose of 50 mg/kg, has the potential to serve as a novel agent for relieving OA.

## Figures and Tables

**Table 1:** Effects of LI73014F2 on the hind paw weight-bearing distribution for 21 days in MIA-induced osteoarthritis in rats. The data are presented as mean ± SEM (*n* = 8/group). * *p* < 0.05, ** *p* < 0.01, compared to the MIA-induced control group; ^##^ *p* < 0.01, compared to the non-MIA-induced control group.

Treatment	Weight Bearing Distribution (%)			
	Day 0	Day 7	Day 14	Day 21
Normal Control	50.91 ± 0.95	49.62 ± 0.60	50.58 ± 0.82	48.56 ± 0.49
MIA Control	31.32 ± 2.84 ^##^	29.19 ± 3.52 ^##^	34.53 ± 1.51 ^##^	39.39 ± 1.23 ^##^
LI73014F2 25 mg/kg	31.13 ± 2.87	37.40 ± 2.43	38.39 ± 1.29	42.67 ± 1.02 *
LI73014F2 50 mg/kg	32.21 ± 3.27	38.90 ± 2.97 *	40.10 ± 1.90 *	43.19 ± 0.96 *
LI73014F2 100 mg/kg	32.04 ± 2.78	38.20 ± 1.85 *	40.06 ± 1.78 *	44.95 ± 0.89 **
Ibuprofen 20 mg/kg	31.32 ± 3.08	36.39 ± 2.95	41.68 ± 1.08 **	45.35 ± 0.66 **

**Table 2:** Effects of LI73014F2 on arthritis index (AI) for 21 days in MIA-induced osteoarthritis in rats. The data are presented as mean ± SEM (*n* = 8/group). ** *p* < 0.01, compared to the MIA-induced control group; ^##^ *p* < 0.01, compared to the non-MIA-induced control group.

Treatment	Arthritis Index (AI)			
	Day 0	Day 7	Day 14	Day 21
Normal Control	0.00	0.00	0.00	0.00
MIA Control	2.00 ± 0.31 ^##^	2.06 ± 0.27 ^##^	1.54 ± 0.05 ^##^	1.50 ± 0.11 ^##^
LI73014F2 25 mg/kg	1.95 ± 0.20	1.70 ± 0.18	1.00 ± 0.07 **	0.74 ± 0.10 **
LI73014F2 50 mg/kg	2.00 ± 0.21	1.51 ± 0.24	1.04 ± 0.10 **	0.74 ± 0.10 **
LI73014F2 100 mg/kg	2.05 ± 0.17	1.48 ± 0.15	0.99 ± 0.11 **	0.71 ± 0.10 **
Ibuprofen 20 mg/kg	1.95 ± 0.19	1.60 ± 0.28	1.04 ± 0.07 **	0.71 ± 0.11 **
